# Probing the Structural, Electronic, and Magnetic Properties of Ag_*n*_V (*n* = 1–12) Clusters

**DOI:** 10.1186/s11671-017-2394-0

**Published:** 2017-12-16

**Authors:** Ran Xiong, Dong Die, Lu Xiao, Yong-Gen Xu, Xu-Ying Shen

**Affiliations:** School of Science, Xihua University, Chengdu, 610039 China

**Keywords:** Ag_*n*_V cluster, Growth behavior, Spectrum, Electronic and magnetic property

## Abstract

The structural, electronic, and magnetic properties of Ag_*n*_V (*n* = 1–12) clusters have been studied using density functional theory and CALYPSO structure searching method. Geometry optimizations manifest that a vanadium atom in low-energy Ag_n_V clusters favors the most highly coordinated location. The substitution of one V atom for an Ag atom in Ag_*n* + 1_ (*n* ≥ 5) cluster modifies the lowest energy structure of the host cluster. The infrared spectra, Raman spectra, and photoelectron spectra of Ag_*n*_V (*n* = 1–12) clusters are simulated and can be used to determine the most stable structure in the future. The relative stability, dissociation channel, and chemical activity of the ground states are analyzed through atomic averaged binding energy, dissociation energy, and energy gap. It is found that V atom can improve the stability of the host cluster, Ag_2_ excepted. The most possible dissociation channels are Ag_*n*_V = Ag + Ag_*n* − 1_V for *n* = 1 and 4–12 and Ag_*n*_V = Ag_2_ + Ag_*n* − 2_V for *n* = 2 and 3. The energy gap of Ag_*n*_V cluster with odd *n* is much smaller than that of Ag_*n* + 1_ cluster. Analyses of magnetic property indicate that the total magnetic moment of Ag_*n*_V cluster mostly comes from V atom and varies from 1 to 5 *μ*_B_. The charge transfer between V and Ag atoms should be responsible for the change of magnetic moment.

## Background

In the past decades, silver clusters have drawn special attention because of their unusually optical and catalytic properties [1–20]. Simultaneously, theoretical and experimental investigations have revealed that an atom doped into a small cluster of another element can fundamentally change the nature of the host cluster [21–44]. Silver clusters doped with different atoms have been expected to tailor the desired optical, electronic, and magnetic properties for potential applications in imaging, sensing, biology, medicine, and nanotechnology [45–55]. For instance, Si doping into silver cluster leads to a broadening and damping of the peaks of UV-visible absorption spectra of Ag clusters [45]. The optical character of Ag_*n*_Au_*m*_ can be adjusted by changing the ratio of silver atoms to gold atoms and Au_4_Ag_4_ might be a potentially promising molecular photoelectric device [46]. In contrast with silver clusters, the binary Ag-Au cluster-modified TiO_2_ electrode improves short-circuit current density and maximum power conversion efficiencies of solar cell [47]. The adsorption energies of a set of typical ligands (−COOH, −CN, −OH, −SH, −CH_3_, −NO_2_, −NH_3_, −NO) are smaller on Ag_12_Au cluster than on Ag_13_ cluster [48]. Ag-Cu nanoalloy is a potential candidate to substitute noble Pt-based catalyst in alkaline fuel cells [49]. The electrons in outer atoms of Ag_12_Cu cluster have a more active characteristic than that of Ag_13_ cluster [50]. The catalytic activity of Ag-Pd alloy cluster for hydrogen dissociation is closely associated with the stoichiometry. The Ag_6_Pd_2_ is the most efficient cluster for hydrogen molecule adsorption and can serve as a promising candidate for H_2_ storage [51]. The introduction of a single 3d transition-metal atom effectively solved the instability problem of the Ag_12_ icosahedron [52]. Recently, several investigations have been carried out for V-doped silver clusters on account of their unique physical and chemical properties [56–59]. Zhang et al. reported that the neutral Ag_12_V cluster show larger relative binding energies compared with pure icosahedral Ag_13_ cluster [56]. Chen et al. found that Pyridine on V@Ag_12_^−^ clusters exhibits the strongest chemical enhancement with a factor of about a thousand [57]. Medel et al. explored the nature of valence transition and spin moment in Ag_*n*_V^+^ clusters that have an enhanced stability for *n* = 14 [58]. However, there are relatively few works concerning the neutral V-doped silver clusters. In particular, the various spectra of Ag_*n*_V clusters have not been obtained but would be extremely helpful for the identification of cluster structure. The structural motif of V-doped silver clusters is also needed to be further explored. The change of magnetic moment of magnetic impurity embedded in a nonmagnetic host still is not fully understood. Accordingly, in the present paper, the geometrical, electronic, and magnetic properties of Ag_*n*_V (*n* = 1–12) clusters will be systematically researched through density functional theory (DFT). It is hoped that this work can provide a reference for understanding the relationship between the function and structure of materials and for related experiments.

## Methods

The accuracy of distinct exchange-correlation functionals, as implemented in GAUSSIAN09 program package (Frisch, M. J. et al., Wallingford, KY, USA) [60], was first verified by calculations on Ag_2_ dimer. The calculated results based on PW91PW91/LanL2DZ (Perdew, J. P. et al., New Orleans, Louisiana, USA) level are in good agreement with experimental findings [61, 62], as summarized in Table 1. On the other hand, test calculations using the different DFT functionals were performed for AgV dimer. Five functionals listed in Table 1 favor the same spin configurations. Thus, this level of theory is used for geometry optimizations and frequency analyses of Ag_*n*_V clusters. A great many initial configurations of Ag_*n*_V clusters were constructed by using CALYPSO which is an efficient structure prediction method [63]. In this method, structural evolution is achieved by particle swarm optimization (PSO) that is a population-based stochastic optimization technique. The bond characterization matrix technique is utilized to enhance searching efficiency and remove similar structures. The significant feature of CALYPSO requires only chemical compositions for a given cluster to predict its structure. Due to the spin polarization effect, each initial structure was optimized at possible spin states. If an imaginary vibrational frequency is found, a relaxation of the unstable structure will be done until the local minimum is really obtained. In all computations, the convergence thresholds were set to 6.0 × 10^−5^ Å for the displacement, 1.5 × 10^−5^ Hartree/Bohr for the forces and 10^−6^ Hartree for a total energy.

**Table 1:** The bond length and electronic properties of Ag_2_ and V_2_ dimers

Dimer	Functional/basis set	R(Å)		D_e_(eV)		VIP(eV)		EA(eV)		*f*(cm^−1^)	
		Calc.	Expt.	Calc.	Expt.	Calc.	Expt.	Calc.	Expt.	Calc.	Expt.
Ag_2_	PW91PW91/LanL2DZ	2.58	2.53^a^	1.78	1.65^a^	7.96	7.65^a^	0.97	1.02^a^	187.0	192.4^a^
	PBEPBE/LanL2DZ	2.59		1.76		7.89		0.92		184.2	
	BP86/LanL2DZ	2.58		1.75		8.05		1.08		188.4	
	LSDA/LanL2DZ	2.50		2.35		8.87		1.52		215.2	
	B3LYP/LanL2DZ	2.61		1.55		7.80		0.93		177.0	
V_2_	PW91PW91/LanL2DZ	1.78	1.77^b^	2.75	2.47 ± 0.22^b^	6.46	6.35^b^	0.46		657.3	

^a^Ref. [67]

^b^Ref. [68]

## Results and Discussions

### Geometrical Structures and Vibrational Spectra

**Fig. 1:**
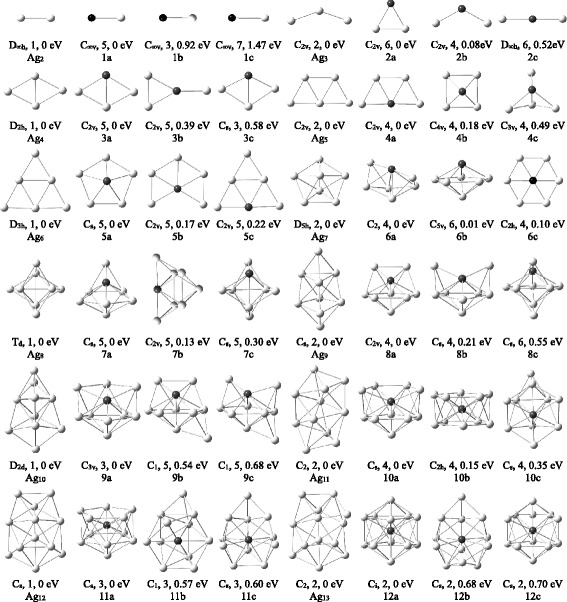
The ground state structures of Ag_*n* + 1_ and Ag_*n*_V (*n* = 2–12) clusters. Two low-lying isomers for Ag_*n*_V clusters. The symmetry, spin multiplicity, and energy difference are given below them. The gray and black balls denote Ag and V atoms, respectively

For Ag_*n*_V (*n* = 1–12) clusters, an extensive structural search has been performed and many isomers have been obtained. The most stable structure and two low-lying isomers for each Ag_*n*_V cluster are displayed in Fig. 1. According to the energies from low to high, these isomers are denoted by na, nb, and nc, where *n* represents the number of Ag atoms in Ag_*n*_V cluster. Their symmetry, spin multiplicity, and energy difference compared to each of the most stable structures are also indicated in the figure. Some physical parameters of the ground state Ag_*n*_V clusters are gathered in Table 2. Meanwhile, in order to examine the effects of dopant V on silver clusters, geometry optimizations of Ag_*n*_ (*n* = 2–13) clusters have been accomplished using the same method and basis set. The lowest energy structures of Ag_*n*_ clusters plotted in Fig. 1 agree well with earlier report [39].

**Table 2:** The dipole moment (*μ*), polarizability $\left({a}_{xx},\kern0.5em {a}_{yy},\kern0.5em {a}_{\mathrm{zz}},\kern0.5em \overline{a}\right)$, zero-point energy (ZPE) and maximum and minimum bond lengths (R_max_, R_min_) of the most stable Ag_*n*_V (*n* = 1–12) clusters and coordination number and average coordination bond length (R_v_) for V atom

Clusters	*μ* (D)	*a*_*xx*_ (a.u.)	*a*_*yy*_ (a.u.)	*a*_zz_ (a.u.)	$\overline{a}$(a.u.)	ZPE(eV)	N	R_max_ (Å)	R_min_ (Å)	R_v_(Å)
AgV	2.07	100.40	100.40	155.87	118.89	0.01	1	2.61	2.61	2.61
Ag_2_V	0.89	124.35	182.31	196.44	167.70	0.03	2	2.73	2.72	2.73
Ag_3_V	1.42	135.44	277.87	178.70	197.34	0.04	3	2.77	2.71	2.76
Ag_4_V	0.82	132.08	340.88	230.06	234.34	0.06	4	2.79	2.70	2.73
Ag_5_V	0.62	327.29	292.90	165.77	261.99	0.08	5	2.82	2.70	2.74
Ag_6_V	0.74	332.60	325.89	245.02	301.17	0.10	6	2.91	2.72	2.77
Ag_7_V	0.21	391.20	340.25	258.36	329.94	0.12	7	3.02	2.73	2.80
Ag_8_V	0.35	417.09	378.36	276.02	357.16	0.14	8	2.88	2.77	2.79
Ag_9_V	0.41	423.10	423.10	300.90	382.37	0.16	9	2.94	2.75	2.80
Ag_10_V	0.77	424.83	364.25	451.18	413.42	0.18	10	3.01	2.76	2.79
Ag_11_V	0.59	402.07	440.99	442.18	428.41	0.20	11	3.13	2.75	2.77
Ag_12_V	0	440.39	439.34	441.45	440.39	0.22	12	3.04	2.76	2.77

The optimized results for AgV dimer show that the quintet spin state is energetically lower than the triplet and septet spin states by 0.92 and 1.47 eV, respectively. Therefore, the quintet AgV is the ground state structure. The most stable structure of Ag_2_V cluster is the triangular 2a with C_2v_ symmetry. The 2a configuration in quartet spin state becomes the 2b isomer. The 3a and 4a isomers, which resemble the lowest energy structures of Ag_4_ and Ag_5_ clusters, are the ground state of Ag_3_V and Ag_4_V clusters. The ground state structure of Ag_4_V cluster is also in accord with the result of Medel et al. [58]. The 4b isomer with V atom on the top is a square pyramid and the first three-dimensional (3D) structure. The 4c isomer possesses a triangular bipyramid structure, and its total energy is above the 4a isomer by 0.49 eV. Other planar and 3D isomers are less stable than 4c isomer.

Starting from *n* = 5, the lowest energy structures of Ag_*n*_V clusters prefer 3D configurations. To prevent from leaving out the ground state, we had also utilized the optimized strategies of substituting an Ag by one V atom from the stable silver cluster or adding Ag atom(s) to small Ag_*n*_V clusters. The 5a and 6a isomers are the most stable structures of Ag_5_V and Ag_6_V clusters. The two isomers are obtained by distorting the geometry from C_5v_ and C_2v_ to C_s_ and C_2_ point groups, respectively. The 6a isomer is 0.62 eV lower in quartet spin state than in sextet spin state. The 5c and 6b isomers are similar to the ground state structures of pure Ag_6_ and Ag_7_ clusters. The 6b isomer is almost degenerate with the 6a isomer. Owing to the Jahn–Teller effect, the planar 6c isomer with C_2h_ symmetry has a slight deviation from D_2h_ symmetry.

With regard to Ag_*n*_V (*n* = 7–12) clusters, the number of isomers increases rapidly with the increase of cluster size. The optimized structures indicate that the energies of Ag_*n*_V clusters with the same configuration increase with the decrease of the coordination number of V atom. As a result, various Ag_*n*_V isomers where V atom occupies the position with the highest coordination number were considered further to make sure that the most stable structures are the global minimum. The lowest energy structures of Ag_7_V, Ag_8_V, Ag_9_V, Ag_10_V, Ag_11_V, and Ag_12_V clusters are 7a, 8a, 9a, 10a, 11a, and 12a in Fig. 1, respectively. Their geometries are qualitatively in accord with results of Medel et al. [58]. These structures are entirely different from the ground state structure of the corresponding Ag_*n* + 1_ clusters and contain a pentagonal bipyramid. The Ag_*n*_V isomers which correspond to the lowest energy structures of Ag_*n* + 1_ clusters lay above each of the ground state structures (na). In addition, the 10b and 12a have a slight deviation from D_5d_ and D_3d_ symmetry. The cage configuration of Ag_12_V cluster, where V atom occupies the central position, is discovered only in the lowest spin states.

**Fig. 2:**
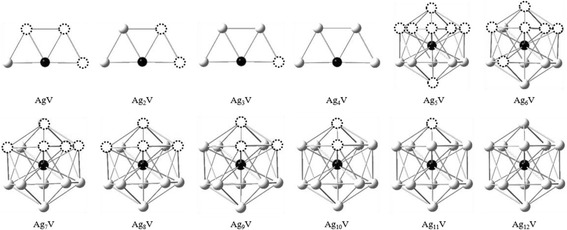
The growth diagram of Ag_*n*_V (*n* = 1–12) clusters

From the optimized results, it is found that the Ag_*n*_V clusters have an obvious growth law. The trapezoid and icosahedron are two basic frameworks for the growth process of Ag_*n*_V cluster, as shown in Fig. 2. The two- to three-dimensional structural transition for Ag_*n*_V cluster occurs at *n* = 5. The transition size of Ag_*n*_V cluster is smaller than that of pure Ag clusters (*n* = 6). For *n* = 5–12, the ground states of Ag_*n*_V clusters are obviously distinct from those of the Ag_*n* + 1_ clusters. The V atom in Ag_*n*_V cluster tends to occupy the most highly coordinated position and is gradually encapsulated in the center by the Ag atoms. This may be attributed to the principle of maximum overlap in chemical bond theory of complexes. Because Ag and V atoms have more orbital overlap under the above circumstances, the energy of Ag_*n*_V cluster, which is also related to the arrangement of Ag atoms, will be lower and then the corresponding cluster is more stable.

**Fig. 3:**
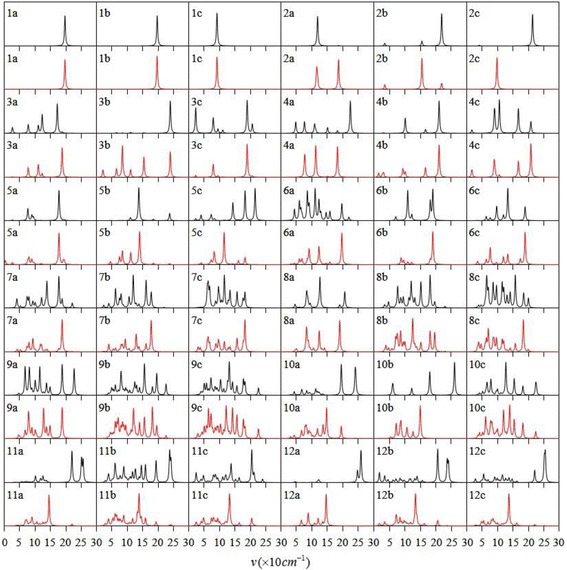
The infrared spectra (black) and Raman spectra (red) of the ground state and two low-lying isomers of Ag_*n*_V (*n* = 1–12) clusters

The infrared and Raman spectroscopy are powerful tools for the identification of cluster structure and material component. Generally, the structural identification is accomplished by comparing experimental findings with theoretical predictions which is an indispensable part. Accordingly, the infrared spectra and Raman spectra of the most stable Ag_*n*_V (*n* = 1–12) clusters are displayed in Fig. 3. The infrared spectrum shows asymmetric vibrations of polar group. Raman spectrum reveals the symmetric vibrations of nonpolar group and skeleton. The AgV dimer have the same infrared and Raman spectra. For other Ag_*n*_V clusters, the strong absorption location of infrared spectrum has a weak peak in Raman scattering spectrum. On the contrary, the Raman scattering peak is strong and the infrared absorption is weak. The peak position in the two kinds of spectra for all isomers are in the range of 15~270 cm^−1^. The most intense peak in the infrared spectrum of each Ag_*n*_V clusters is related to the Ag-V stretching vibration.

### Electronic Properties

**Fig. 4:**
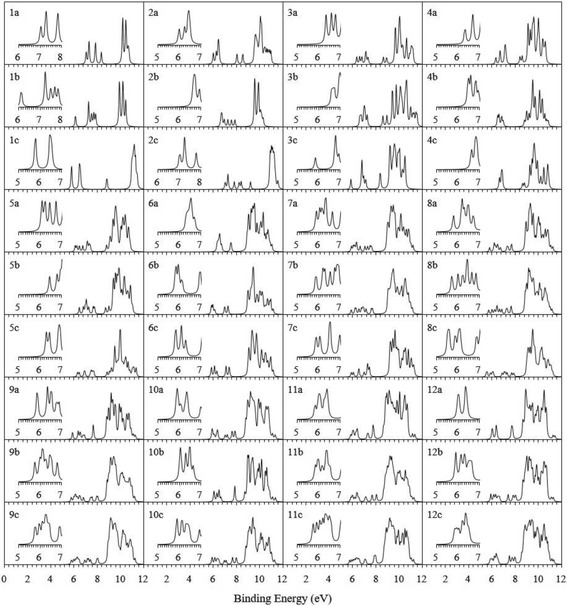
Simulated PES of the ground state and two low-lying isomers of Ag_*n*_V (*n* = 1–12) clusters

The vertical ionization potential (VIP) and electron affinity (EA) are two primary quantities to probe the electronic properties and can be calculated as follows:

1 $$ \mathrm{VIP}=E\left(\mathrm{cationic}\  \mathrm{cluster}\right)-E\left(\mathrm{cluster}\right) $$

2 $$ \mathrm{EA}=E\left(\mathrm{cluster}\right)-E\left(\mathrm{anionic}\  \mathrm{cluster}\right) $$

where *E*(cationic cluster) and *E*(anionic cluster) are the single-point energies of cationic and anionic clusters in the geometry of neutral cluster. For the lowest energy Ag_*n* + 1_ and Ag_*n*_V clusters, Table 3 lists the calculated VIP, EA, and the available experimental values. The calculated VIPs and EAs of Ag_*n* + 1_ clusters are in line with their measured data. This consistency confirms the reliability of the current theoretical approach again. Moreover, we note that AgV dimer has the biggest VIP and the smallest EA. This implies that AgV is hard to lose or require an electron. The icosahedral Ag_12_V cluster has the biggest EA and is easy to get one more electron. To offer reference material for photoelectron spectroscopy experiment in the aftertime, the theoretical photoelectron spectra (PES) of the ground state and two low-lying structures of Ag_*n*_V (*n* = 1–12) clusters were simulated by adding the first VIP to each occupied orbital energy relative to the HOMO and fitting them with a Lorentz expansion scheme and a broadening factor of 0.1 eV, as shown in Fig. 4. The distribution of energy level of these clusters is in the range of 5.5 to 12 eV. The experimenters can make use of the PES spectra to distinguish these clusters.

**Table 3:** VIP and VEA of the ground state Ag_*n* + 1_ and Ag_*n*_V clusters. The data in parentheses are experimental findings

Clusters	VIP(eV)	VEA(eV)	Clusters	VIP(eV)	VEA(eV)
Ag_2_	7.96	0.97	AgV	7.04	0.82
Ag_3_	6.92(6.20^a^)	2.17	Ag_2_V	5.99	1.28
Ag_4_	6.60(6.65^a^)	1.63	Ag_3_V	6.35	1.49
Ag_5_	6.28(6.35^a^)	2.04	Ag_4_V	6.33	1.86
Ag_6_	7.15(7.15^a^)	1.33	Ag_5_V	6.09	1.47
Ag_7_	6.06(6.40^a^)	1.94	Ag_6_V	6.32	1.87
Ag_8_	6.99	1.17	Ag_7_V	5.89	1.69
Ag_9_	6.01	2.27	Ag_8_V	5.79	1.87
Ag_10_	5.95	1.66	Ag_9_V	5.87	2.08
Ag_11_	5.86	2.42	Ag_10_V	5.88	2.24
Ag_12_	6.13	2.09	Ag_11_V	5.83	2.31
Ag_13_	5.61	2.36	Ag_12_V	5.99	2.45

^a^Ref. [67]

**Fig. 5:**
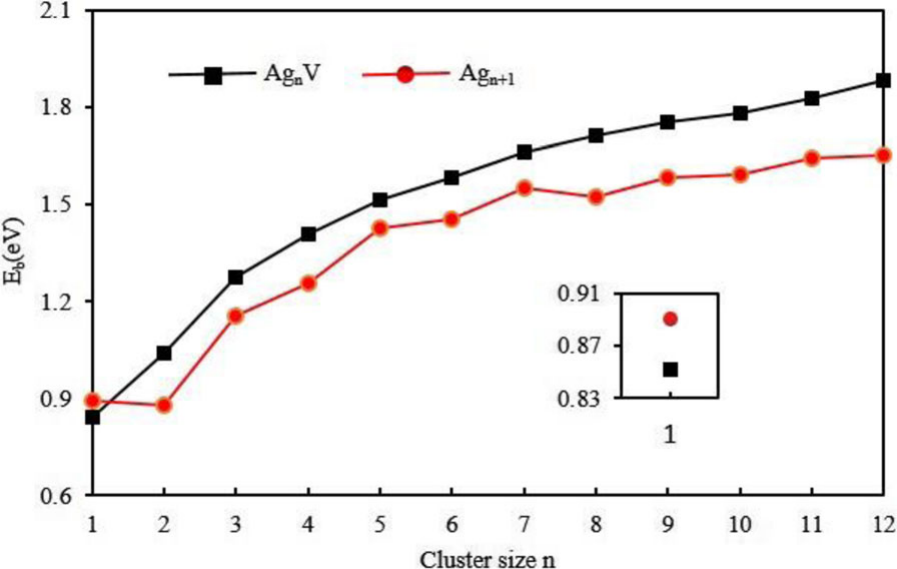
The averaged binding energies of the lowest energy Ag_*n* + 1_ and Ag_*n*_V (*n* = 1–12) clusters

In order to examine the influence of V atom on the stability of silver clusters, the atomic averaged binding energies (*E*_b_) of the most stable Ag_*n* + 1_ and Ag_*n*_V clusters can be estimated as follows:

3 $$ {E}_b\left({\mathrm{Ag}}_{n+1}\right)=\left[\left(n+1\right)E\left(\mathrm{Ag}\right)-E\left({\mathrm{Ag}}_{n+1}\right)\right]/\left(n+1\right), $$

4 $$ {E}_{\mathrm{b}}\left({\mathrm{Ag}}_n\mathrm{V}\right)=\left[ nE\left(\mathrm{Ag}\right)+E\left(\mathrm{V}\right)-E\left({\mathrm{Ag}}_n\mathrm{V}\right)\right]/\left(n+1\right), $$

where *E*(Ag), *E*(Ag_*n* + 1_), *E*(V)_,_ and *E*(Ag_*n*_V) are the energies of Ag atom, silver cluster, V atom, and Ag_n_V cluster, respectively. The calculated binding energies per atom for the most stable Ag_*n* + 1_ and Ag_*n*_V clusters are plotted in Fig. 5. It is clear from this figure that the *E*_*b*_ of Ag_*n*_V cluster is a monotonically increasing function of the cluster size and larger than that of Ag_*n* + 1_ cluster for *n* ≥ 2. Especially, the *E*_*b*_ of doped cluster increase rapidly for the planar structures and gradually for the 3D structures. This means that the bonding force among atoms becomes stronger and stronger in the process of growth. The substitution of a V atom for an Ag atom in Ag_*n* + 1_(*n* ≥ 2) clusters can evidently enhance the stability of the host clusters. On the other hand, the bond energy of diatomic cluster should be closely related to the bond length. The *E*_b_ of AgV dimer is smaller than that of Ag_2_. The abnormal change may be ascribed to the fact that the bond distance of AgV (2.61 Å) is longer than that of Ag_2_ (2.58 Å).

The thermal stability of clusters can be examined by the dissociation energy (DE), which is different for the distinct dissociation channels. The most basic dissociation channel is the splitting of a larger cluster into two smaller clusters. The corresponding DE is small relative to other dissociation channel. Hence, the subsequent dissociation channels are investigated for the most stable Ag_*n*_V (*n* = 1–12) clusters.

5 $$ {\mathrm{Ag}}_n\mathrm{V}\to {\mathrm{Ag}}_m+{\mathrm{Ag}}_{n-m}\mathrm{V} $$

where *m* is not more than *n*. The DEs of the above dissociation channels are defined as follows:

6 $$ {\mathrm{DE}}_m\left({\mathrm{Ag}}_n\mathrm{V}\right)=E\left({\mathrm{Ag}}_m\right)+E\left({\mathrm{Ag}}_{n-m}\mathrm{V}\right)-E\left({\mathrm{Ag}}_n\mathrm{V}\right) $$

where *E* represents the energy of the corresponding cluster or atom. The DEs of Ag_*n*_V clusters for the different dissociation channels have been listed in Table 4. The small DE indicates that corresponding dissociation channel is easy to take place. That is to say, the dissociation channel corresponding to the minimum DE is most likely to occur. It can be seen from Table 4 that the most preferred dissociation channels of Ag_*n*_V clusters are Ag_*n*_V = Ag + Ag_*n* − 1_V for *n* = 1 and 4–12 and Ag_*n*_V = Ag_2_ + Ag_*n* − 2_V for *n* = 2 and 3. The minimum DE (2.54 eV) of Ag_12_V cluster is biggest in all doped cluster, implying that the icosahedral cluster is more stable than other cluster. In addition, we find that the change trend of the minimum DE of the 3D neutral Ag_*n*_V (*n* = 5–12) cluster is the same as that of abundances of the cationic Ag_*n*_V^+^ cluster [64, 65]. However, there is no such relationship between planar Ag_*n*_V and Ag_*n*_V^+^ for *n* = 2–4.

**Table 4:** The dissociation energy (D_E_, eV) of Ag_*n*_V clusters for the distinct dissociation channels

Ag_*n*_V clusters dissociation channel	D_E_ *n* = 1	D_E_ *n* = 2	D_E_ *n* = 3	D_E_ *n* = 4	D_E_ *n* = 5	D_E_ *n* = 6	D_E_ *n* = 7	D_E_ *n* = 8	D_E_ *n* = 9	D_E_ *n* = 10	D_E_ *n* = 11	D_E_ *n* = 12
AgV = Ag_*n*_ + Ag_1 − *n*_V	1.70											
Ag_2_V = Ag_*n*_ + Ag_2 − *n*_V	1.43	1.36										
Ag_3_V = Ag_*n*_ + Ag_3 − *n*_V	1.98	1.63	2.48									
Ag_4_V = Ag_*n*_ + Ag_4 − *n*_V	1.94	2.13	2.72	2.44								
Ag_5_V = Ag_*n*_ + Ag_5 − *n*_V	2.05	2.21	3.33	2.79	2.82							
Ag_6_V = Ag_*n*_ + Ag_6 − *n*_V	1.99	2.26	3.35	3.35	3.10	2.53						
Ag_7_V = Ag_*n*_ + Ag_7 − *n*_V	2.22	2.43	3.63	3.59	3.89	3.05	3.14					
Ag_8_V = Ag_*n*_ + Ag_8 − *n*_V	2.10	2.54	3.68	3.75	4.02	3.72	3.54	3.14				
Ag_9_V = Ag_*n*_ + Ag_9 − *n*_V	2.15	2.47	3.84	3.86	4.23	3.90	4.26	3.45	3.84			
Ag_10_V = Ag_*n*_ + Ag_10 − *n*_V	2.03	2.40	3.65	3.90	4.21	3.99	4.31	4.05	4.17	3.75		
Ag_11_V = Ag_*n*_ + Ag_11 − *n*_V	2.35	2.60	3.90	4.02	4.57	4.29	4.72	4.42	5.09	4.39	4.41	
Ag_12_V = Ag_*n*_ + Ag_12 − *n*_V	2.54	3.11	4.29	4.46	4.89	4.84	5.21	5.02	5.65	5.50	5.24	4.80

**Fig. 6:**
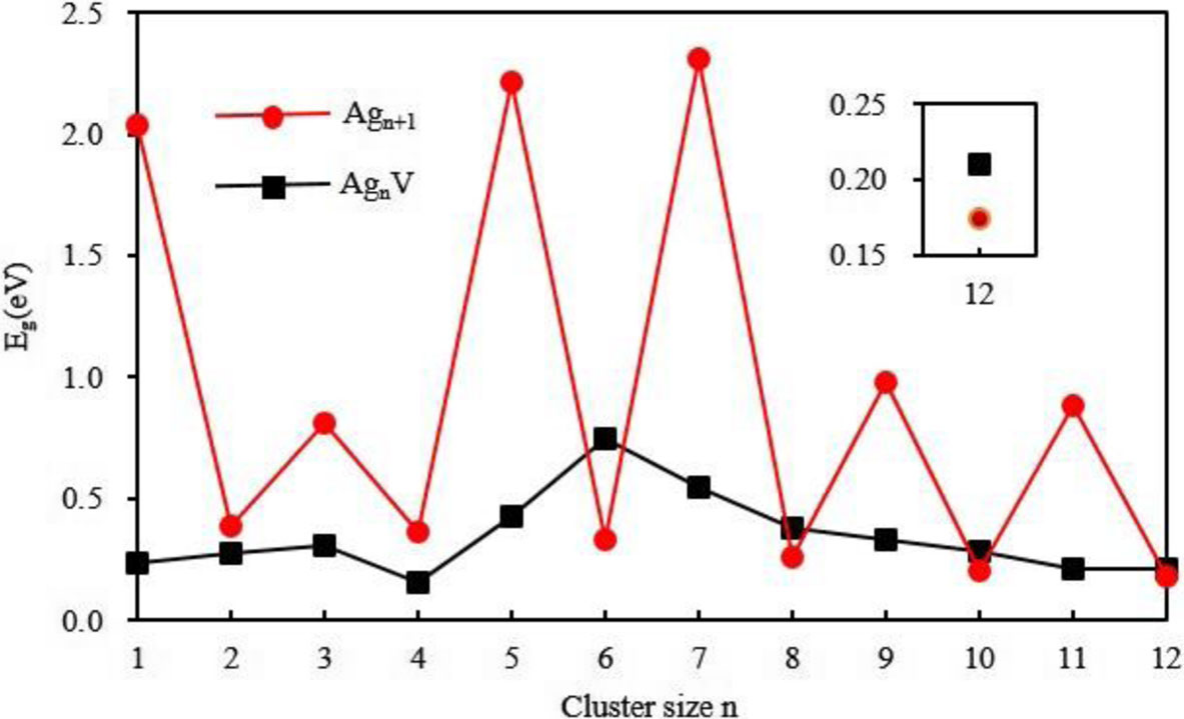
The HOMO-LUMO energy gaps of the ground state Ag_*n* + 1_ and Ag_*n*_V (*n*=1–12) clusters

The energy gap (*E*_g_) between the highest occupied molecular orbital (HOMO) and lowest unoccupied molecular orbital (LUMO) is always considered to be an important quantity that characterizes the chemical activity of the small metal clusters. A large energy gap is related to a high chemical stability. For the ground state Ag_*n* + 1_ and Ag_*n*_V clusters, Fig. 6 shows the energy gaps as a function of the cluster size. An odd-even alternation is observed in the energy gaps of pure silver clusters. This alternation can be explained by the electron pairing effect, i.e., the electron shielding effect of two electrons occupying the same HOMO is much smaller than that of two electrons occupying different orbits. An Ag atom ([Kr]4f^14^4*d* ^10^5*s* ^1^) in Ag_*n* + 1_ cluster is substituted by a V ([Ar]3*d* ^3^4*s* ^2^) atom. For odd *n*, the closed shell of Ag_*n* + 1_ cluster is replaced by the open shell of Ag_*n*_V cluster. Of course, the *E*_g_ of Ag_*n*_V cluster with odd *n* is less than that of Ag_*n* + 1_ cluster. This decrease is very obvious. For even *n*, both Ag_*n* + 1_ and Ag_*n*_V clusters have an unrestricted shell. The *E*_g_ should depend on their structures. In this case, we note that the *E*_g_ of Ag_*n*_V (*n* = 2 and 4) cluster with planar structure is smaller than that of Ag_*n* + 1_ cluster and the *E*_g_ of Ag_*n*_V (*n* = 6, 8, 10, and 12) cluster with 3D structure is a little bigger than that of Ag_*n* + 1_ cluster. In general, the substitution of one V atom for an Ag atom in Ag_*n* + 1_ clusters with even *n* has little effect on the energy gap of the host cluster.

### Magnetic Properties

**Fig. 7:**
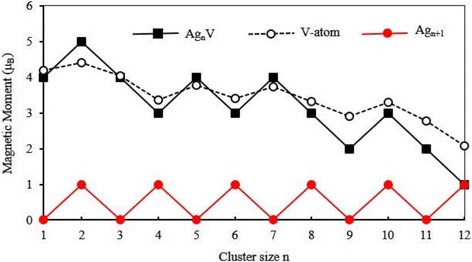
Total magnetic moment of the ground state Ag_*n* + 1_ and Ag_*n*_V (*n* = 1–12) clusters and local magnetic moment on V atom

**Fig. 8:**
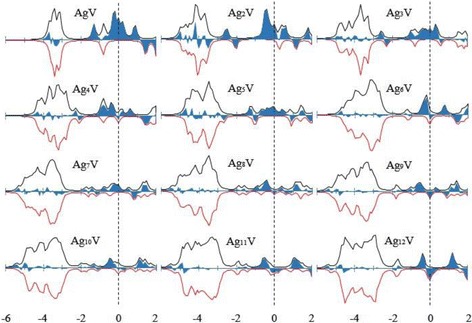
The SDOS of ground state Ag_*n*_V (*n* = 1–12) clusters. Spin up is positive and spin down is negative. A broadening factor δ = 0.1 eV is used. Spin up minus spin down is the blue part. The dashed line indicates the location of the HOMO level

The magnetic property of cluster is frequently used in the preparation of nanoelectronic devices and high-density magnetic storage materials. The total magnetic moment of cluster consists of the spin magnetic moment and orbital magnetic moment of electrons. The spin magnetic moment of an electron is much greater than the orbital magnetic moment, and thereby, the magnetic moment of cluster is dominated by the spin magnetic moment. The total magnetic moment of the lowest energy Ag_*n*_V clusters (*n* = 1–12) clusters has been calculated and are presented in Fig. 7, where we have also plotted the total magnetic moment of the host clusters. The magnetic moments of the most stable Ag_*n* + 1_ clusters are completely quenched for odd *n* and are 1 μ_B_ for even *n*. The small Ag_*n*_V clusters have a large magnetic moment. With the increase of the cluster size, the magnetic moment of Ag_*n*_V clusters decreases in waves. When *n* = 12, the Ag_12_V has the same magnetic moment as Ag_13_ cluster. This means that the doping of V atom can only enhance the magnetism of small silver clusters. As an effort to account for the magnetism, Fig. 8 shows the spin density of states (SDOS) for the ground state Ag_*n*_V clusters. It is obvious from this figure that the Ag_*n*_V clusters have some magnetic domains which decrease with the increase of clusters size. All the lowest energy structures have a strong band between − 5 eV and − 2.5 eV, which is composed mainly of the valence *s* and *d* orbitals of the Ag and V atoms. The energy levels near the HOMO, *E* − *E*_HOMO_ =  − 1~0 eV, act as a key role in the determination of magnetic behavior of Ag_*n*_V clusters.

To explore the magnetic properties further, we have carried out the natural bond orbital analysis for the most stable Ag_*n*_V clusters [66]. The local magnetic moments on V atom are 4.18 *μ*_B_ for AgV, 4.41 *μ*_B_ for Ag_2_V, 4.03 *μ*_B_ for Ag_3_V, 3.36 *μ*_B_ for Ag_4_V, 3.78*μ*_B_ for Ag_5_V, 3.40 *μ*_B_ for Ag_6_V, 3.73 *μ*_B_ for Ag_7_V, 3.33 *μ*_B_ for Ag_8_V, 2.91 *μ*_B_ for Ag_9_V, 3.29 *μ*_B_ for Ag_10_V, 2.77 *μ*_B_ for Ag_11_V, and 2.08 *μ*_B_ for Ag_12_V, as shown in Fig. 7. Overall, the magnetic moment of V atom gradually decreases with the size of clusters increasing. The magnetic moment provided by Ag atoms is very small. Furthermore, except for Ag_2_V, Ag_5_V, and Ag_7_V clusters, the total magnetic moment of Ag atoms in other doped clusters exhibit the antiferromagnetic alignment with respect to the V atom’s magnetic moment. In other words, the total magnetic moments of all Ag_*n*_V clusters are chiefly derived from the paramagnetic V atom, as shown in Fig. 7.

**Fig. 9:**
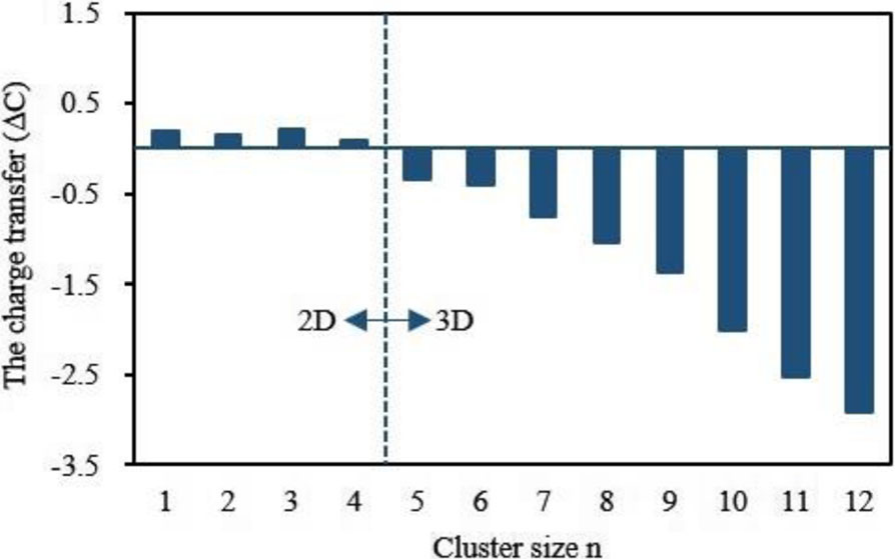
The charge transfer of V atom in the most stable Ag_*n*_V (*n* = 1–12) clusters. Free V atom as the reference point

**Fig. 10:**
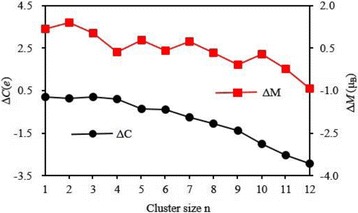
The charge transfer (ΔC) and the change of magnetic moment (ΔM) of V atom in the most stable Ag_*n*_V (*n* = 1–12) clusters

The local magnetic moment and charge on 4*s*, 3*d*, 4*p*, and 4*d* shells of V atom in the lowest energy Ag_*n*_V cluster are listed in Table 5. One can be seen from this table that the partially occupied 3*d* shell play a crucial role in determining the magnetism of V atom and its magnetic moment is 2.01~3.82 *μ*_B_. The 4*s* and 4*p* shells, which are nonmagnetic for a free V atom, produce a little of the magnetic moment. The 4*d* shell is almost non-magnetic. The charge on 3*d* and 4*p* shells increases by 0.77–1.97 and 0.03–2.41 *e* respectively. Especially, the charge on the 4*p* orbital increases with the increase of the clusters size. A very few charge is found on the 4*d* orbit of V atom in Ag_*n*_V (*n* = 4–12) cluster. Nevertheless, the charge on 4*s* shell reduces by 1.02–1.54 *e*. The charge transfer hints that V atom in Ag_*n*_V clusters has a hybridization among *s*, *p*, and *d* shells. As we know, the isolated V atom has five valence electrons. At the same time, the charge of V atom in Ag_*n*_V cluster can be obtained from Table 5. From the principle of charge conservation, 0.10–0.21 *e* transfer from V atom to Ag atoms for the planar Ag_*n*_V (*n* = 1–4) clusters, whereas 0.35–2.92 *e* from Ag atoms to V atom for the 3D Ag_*n*_V (*n* = 5–12) clusters, as shown in Fig. 9. If *M* and *C* denote the magnetic moment and valence electron of V atom in Ag_*n*_V clusters, both the variation of magnetic moment (*ΔM* = *M* − 3) and charge transfer (*ΔC* = 5 − *C*) have the same changing trend, as displayed in Fig. 10. It can be concluded from Fig. 10 that charge transfer should be the reason for the modification of the magnetic moment of V atom in Ag_*n*_V clusters.

**Table 5:** The charge (Q) and local magnetic moment (M) of 4*s*, 3*d*, 4*p*, and 5*d* states for the V atom in the ground state Ag_*n*_V clusters

Clusters	4 *s*–V		3*d*–V		4*p*–V		4*d*–V	
	Q(e)	M (μ_B_)	Q(e)	M (μ_B_)	Q(e)	M (μ_B_)	Q(e)	M (μ_B_)
AgV	0.98	0.48	3.79	3.69	0.03	0.01	0	0
Ag_2_V	0.81	0.53	3.92	3.82	0.12	0.06	0	0
Ag_3_V	0.64	0.32	3.90	3.68	0.25	0.03	0	0
Ag_4_V	0.58	0.04	3.77	3.31	0.53	0.01	0.02	0
Ag_5_V	0.49	0.07	4.03	3.65	0.82	0.06	0.01	0.01
Ag_6_V	0.46	0.04	4.00	3.34	0.92	0.02	0.02	0
Ag_7_V	0.47	0.07	4.14	3.56	1.12	0.10	0.02	0
Ag_8_V	0.48	0.04	4.22	3.20	1.33	0.09	0.02	0
Ag_9_V	0.47	0.03	4.34	2.80	1.53	0.07	0.03	0.01
Ag_10_V	0.50	0.06	4.53	3.11	1.94	0.12	0.04	0
Ag_11_V	0.50	0.04	4.74	2.64	2.25	0.09	0.04	0
Ag_12_V	0.50	0.02	4.97	2.01	2.41	0.05	0.04	0

## Conclusions

The structural, electronic, and magnetic properties of Ag_*n*_V (*n* = 1–12) clusters have been investigated on the basis of DFT and CALYPSO structure searching method. The results indicate V atom in the lowest energy Ag_*n*_V cluster tends to occupy the position with the highest coordination number. The substitution of an Ag atom in Ag_*n* + 1_ (*n* ≥ 5) cluster by one V atom changes the geometry of the host clusters. The infrared spectra, Raman spectra, and PES of Ag_*n*_V (*n* = 1–12) clusters are expected to identify the ground states in times to come. Aside from AgV, the stability of other Ag_*n*_V cluster is higher than that of Ag_*n* + 1_ cluster. The relatively easy dissociation channels are Ag_*n*_V = Ag + Ag_n − 1_V for *n* = 1 and 4–12 and Ag_*n*_V = Ag_2_ + Ag_*n* − 2_V for *n* = 2 and 3. The chemical activity of Ag_*n*_V cluster with odd *n* is higher than that of Ag_*n* + 1_ clusters. The magnetic moments of Ag_*n*_V clusters originate mainly from the doped V atom and decrease gradually from 5 to 1 *μ*_B_ with the increase of cluster size. The change of magnetic moment may be attributed to the charge transfer between V and Ag atoms.

## Abbreviations

3D: Three-dimensional
DE: Dissociation energy
DFT: Density functional theory
EA: Electron affinity
HOMO: Highest occupied molecular orbital
LUMO: Lowest unoccupied molecular orbital
PSO: Particle swarm optimization
VIP: Vertical ionization potential
